# The Effect of Social Support on Glycemic Control in Patients with Type 2 Diabetes Mellitus: The Mediating Roles of Self-Efficacy and Adherence

**DOI:** 10.1155/2017/2804178

**Published:** 2017-05-25

**Authors:** Yechang Shao, Lu Liang, Linjing Shi, Chengsong Wan, Shouyi Yu

**Affiliations:** ^1^Guangdong General Hospital, Guangdong Academy of Medical Sciences, Guangzhou, China; ^2^School of Public Health, Southern Medical University, Guangzhou, China; ^3^Division of Life Science, Center for Cancer Research, Hong Kong University of Science and Technology, Clear Water Bay, Hong Kong

## Abstract

Ample evidence suggests that social support, self-efficacy, and adherence significantly, independently, and together affect glycemic control in patients with type 2 diabetes mellitus (T2DM), but the pathway from social support to glycemic control remains unclear. This study hypothesized that the effect of social support on glycemic control was mediated sequentially by self-efficacy and adherence. Patients with T2DM were recruited from two hospitals in Guangzhou, China, from January 1 to July 31, 2014, and their sociodemographic clinical data and their assessments on social support, self-efficacy, and adherence were obtained from medical records and self-completed questionnaires. Of the 532 patients who participated, 35% achieved glycemic control (i.e., HbA1c < 7%). Social support, self-efficacy, and adherence had significant correlations with each other and with glycemic control (*P* < 0.05). Regression analyses and structural equation modeling showed that better social support was associated to better patient self-efficacy, which, in turn, was associated with better medical adherence, which was associated with improved glycemic control, and the relationship between social support and glycemic control was sequentially and completely mediated by self-efficacy and adherence. The five goodness-of-fit indices confirmed that our data fitted the hypothesized pathway model strongly.

## 1. Introduction

Diabetes prevalence has been growing rapidly in recent decades. The number of people with diabetes increased from 108 million in 1980 to 422 million in 2014 globally; in 2012, 3.7 million deaths were attributed to diabetes and high blood glucose [1]. Type 2 diabetes mellitus (T2DM) is the most common diabetes, a chronic and metabolic disease indicated by elevated level of blood glucose due to insulin deficiency and resistance. Controlling glycemic level in T2DM is critical in preventing long-term microvascular and macrovasuclar complications [2–4]. However, the majority of T2DM patients fail to control their level of blood glucose, for various reasons [4–7].

Adherence, defined as the extent to which a patient complies with prescribed treatment under limited supervision, has been recognized as the most important factor in managing many chronic conditions [7]. Typical treatment for T2DM involves a complex regimen of medications, diabetic specific diet, exercises, and self-monitoring of glucose levels, and adherence to such treatment regimen has been repeatedly proven to be pivotal in maintaining proper glycemic control and reducing the risks of complications [8]. But the regimen requires great efforts by the patients who are likely dealing with multiple demands, and a wide variety of social and psychological factors can interrupt and interfere, causing nonadherence [7, 9, 10]. One of the critical factors is patient self-efficacy, defined as the patient's belief and confidence in controlling progresses of his or her medical conditions, which have been demonstrated to significantly affect adherence to treatment regimen and outcomes among patients with T2DM and other chronic illnesses [11, 12].

The importance of social support in T2DM management has also been recognized [13–15], but its role in glycemic control, especially in the context of self-efficacy and adherence, is not fully understood. Social support, a multidimensional concept referring to the support a patient perceives and receives from his or her social network such as family and friends, is usually measured in three dimensions [16]. The first is objective support, also known as practical or visible support, including direct assistance materially from social network of stable social connections (i.e., family, friends, colleagues, and so forth) and unstable connections (i.e., informal support groups, and so forth). The second is subjective support, which refers to the emotional and subjective experience of being respected, supported, and understood. The third dimension is the extent of social support utilization, including access and acceptance to various aspects of support and attempts in seeking support from family, relatives, friends, colleagues, and larger community. Previous studies showed that social support improved self-efficacy for patients in T2DM management [11], and patients from cohesive families tended to be more adherent to medication than patients from families in conflicts [15]. However, the findings on the role of social support in glycemic control are inconsistent. For example, Chlebowy and Garvin showed that there was no significant relationship between social support and HbA1c levels [17], and Fortman et al. found that higher functional social support was related to poorer glycemic control [18]. Mondesir et al. observed that the relationship between social support and glycemic control differed by gender: higher social support was significantly associated with lower levels of HbA1c in elderly male patients, but with higher levels of HbA1c in elderly female patients [6].

Such inconsistent results are likely due to the tangled relationships among some key factors in the pathway from social support to patient outcomes. In this study, we intended to extend the evidence base and improve our understanding of the relationship between social support and glycemic control in T2DM patients. More specifically, we hypothesize that better social support perceived or received by a T2DM patient could become internalized, reinforcing the patient's self-efficacy, which, in turn, improves the patient's adherence to medical regimen, which, in turn, leads to improved glycemic control. In other words, we intended to examine whether the effect of social support on glycemic control was mediated sequentially by self-efficacy and adherence.

## 2. Methods

### 2.1. Setting and Participants

This study was approved by the Guangdong General Hospital Institutional Review Board. The participants were provided with both written and oral information regarding the study, signed informed consent forms, and were informed that they were free to withdraw from the study at any time.

Participants were recruited from the outpatients and inpatients who visited the endocrine clinics of two of the largest hospitals in Guangzhou City, Guangdong Province, between January 1 and July 31, 2014. The inclusion criteria included (a) patients who were diagnosed as having T2DM according to the 2010 American Diabetes Association (ADA) criteria for at least 1 year; (b) those who were at least 18 years of age; (c) those who had no concurrent malignant tumor, type 1 diabetes, gestational diabetes, vision impairment due to complications, limited physical activity due to advanced renal failure, or acute complications; (d) those who were Chinese inhabitants of the city of Guangzhou; and (e) those who were able and willing to complete the questionnaires.

Once a patient was recruited and signed on to participate, the patient's demographic information was collected, including age, gender, education, personal income, and number of family members living together. The patients were then asked to complete the questionnaires containing items on social support, self-efficacy, and adherence. The patient's medical records were then reviewed to obtain further relevant information regarding disease history, medical conditions, treatment, and other biomedical data, and the blood sample was drawn if a patient's medical records had no HbA1C values obtained in the last three months before being recruited to participate in this study.

### 2.2. Measurement and Data Collection

#### 2.2.1. Social Support

We used the Social Support Rating Scale (SSRS) designed by Xiao [16, 19] to collect data on social support. SSRS is the commonly used assessment tool for social support in China. It measures three dimensions of social support: objective support (i.e., actual or visible support, including material direct assistance and social networking), subjective support (i.e., experience or emotional support, referring to an individual's sense of being respected, supported, understood, and/or satisfied in a society), and support utilization (i.e., the extent of accepting help and actively looking for support from family, relatives, friends, colleagues, and community). The SSRS questionnaire contains 10 items scored on Likert scales, with objective support calculated as the total points from items 2, 6, and 7, subjective support calculated as the total points from items 1, 3, 4, and 5, support utilization calculated as the total points from items 8, 9, and 10, and overall social support calculated as the total points across all 10 items (the full questionnaire and scoring method are available from the author upon request). The scores for objective support, subjective support, and support utilization range from 1 to 22, 8 to 32, and 3 to 12, respectively, and the overall social support score ranges from 12 to 66.

#### 2.2.2. Self-Efficacy

We used the self-efficacy scale designed by Lorig et al. in their research of self-management behavior among patients with chronic diseases, including T2DM [20]. The questionnaire contains 6 items that measures multiple aspects of self-efficacy, including emotional control, communication with doctors, symptom management, role function, and perceived adaptability to different aspects of chronic diseases, such as pain, fatigue, and trust. Each item is scored from 0 (no confidence at all) to 9 (full confidence), with the average score of the six items indicating a patient's overall level of self-efficacy. The Cronbach's *α* value of this scale was estimated at 0.89 [20].

#### 2.2.3. Adherence

There has been a great amount of literature on the theory and measurement of medical adherence [21–23], but none of the existing scales fits our purpose and setting precisely. Therefore, we developed an adherence scale according to the treatment principle of diabetes and some previous relevant works. Our scale contains 13 items, organized into three subscales: treatment adherence subscale containing 3 items (Do you take the medicine every day according to the doctor's advice? Do you take the dosages according to the doctor's advice? Do you take the medication on time?); diet adherence subscale containing 6 items (compliance with diet plan, having meal or snack at regular hours, weighing food regularly, following recommendations in food intake, regular intake of sweets, and regular intake of fatty food); and lifestyle adherence subscale containing 4 items (smoking, drinking, regular exercise, and emotional relaxation). Treatment and diet adherence items were scored from 1 to 4 points indicating “hardly” to “complete” adherence, and lifestyle adherence items were scored from 1 to 3 points indicating “never,” “occasionally,” or “often.” The overall adherence and subscales were calculated as the summed points of all included items accordingly, with a higher score indicating a greater level of adherence.

#### 2.2.4. Glycemic Control

Glycemic control was represented by the level of HbA1c, which has an ideal range from 4.0% to 6.7%. Each participating patient's medical records were reviewed, and the last HbA1c assessment over the previous 3 months (if multiple measures exist) was taken as the patient's HbA1c in this study. If a patient had no HbA1c value from the last three months in the medical records, his or her blood sample was collected after the patients completed the questionnaires, and HbA1c was assessed at the lab of the participating hospital.

Based on the 2010 ADA definition and following previous researchers [6, 13, 17, 24], we categorized participating patients into two groups: those with good glycemic control if HbA1c values were <7% and those with poor glycemic control if HbA1c values were ≥7%.

### 2.3. Statistical Analysis

The collected data were entered into a database using EpiData 3.1 software. Simultaneous data entry was carried out by two graduate students hired by the research team, and the consistency check function of the EpiData software was tested to ensure accuracy. Unpaired *t*-test and chi-square test were used to compare basic demographic data between patients whose HbA1c were under and those not under control. Pearson's correlation coefficients were calculated to examine the pairwise associations between scores of social support, self-efficacy, adherence, and HbA1c levels.

We used two approaches to verify our hypothesized pathway from social support to self-efficacy, to adherence, and, finally, to glycemic control. First, we run a series of linear (when dependent variable was continuous) and logistic regressions (when dependent variable was dichotomous), adjusted for the potential confounders (e.g., age, gender, education, monthly income, family size, alcohol consumption, and physical exercise). According to Baron and Kenny [25], mediation is demonstrated when (a) the main independent variable is significantly associated with the dependent variable, (b) the main independent variable is significantly associated with the hypothesized mediating variable, and (c) the hypothesized mediator is significantly associated with the dependent variable when the independent variable is controlled for. If in step (c), the independent variable becomes insignificant, then its effect on the dependent variable is considered to be completely mediated by the mediator; if it is still significant, but its standardized regression coefficient is smaller than that in step (a), then its effect is considered to be partially mediated [26].

To apply this approach to the mediating effect of self-efficacy (SE) on the relationship between social support (SS) and glycemic control (GC), using the hypothesized pathway SS→SE→GC, for example, we run the following three regressions:

(1) $$\begin{array}{c} GC=a+bSS+e1,\\ SE=c+dSS+e2,\\ GC=f+gSS+hSE+e3, \end{array}$$

where *b*, *d*, and *g* are regression coefficients of interest and expressed in standardized coefficients for convenience of comparisons; *a*, *c*, and *f* are intercepts; and e1, e2, and e3 are error terms. SE is deemed to have a complete mediating effect on the relationship between SS and GC if *b*, *d*, and *h* are statistically significant, but *g* is statistically not significant. SE is deemed to have partial mediating effect if *g* is statistically significant but smaller than *b*. Please note that the notations are made simplistically to help describe the deduction process; since GC is dichotomous, the regressions on GC are logistic regressions, while the regression on SE is linear.

The regressions offer straightforward insights into the mediating effects of self-efficacy and adherence on the relationship between social support and glycemic control, but it does not provide straightforward, robust estimates on the goodness-of-fit between the data and the hypothesized pathway. To further confirm our hypothesis, we used structural equation modeling estimated by SPSS AMOS 22.0 (IBM SPSS; SPSS Inc., Armonk, NY, USA). AMOS provides not only the standardized estimates and their standard errors for all the paths hypothesized, using maximum likelihood and bootstrap estimation methods, but also produces a set of indices on the overall goodness-of-fit between the actual data and the specified path model. The goodness-of-fit indices include, but not limited to, the chi-square test, the goodness fit index (GFI), the adjusted goodness-of-fit index (AGFI), the comparative fit index (CFI), and the root mean square error of approximation (RMSEA), and, in general, the hypothesized pathway is deemed fit with the data (i.e., validated by the data) if the chi-square test is small with *P* > 0.05; GFI, AGFI, and CFI > 0.9; and RMSEA ≤ 0.05 [27]. Two-tailed *P* values less than 0.05 were considered statistically significant.

## 3. Results

### 3.1. Participant Characteristics

A total of 532 T2DM patients participated in the study. Most patients were elderly, female, living in 2-3 people households. Mean HbA1c values of these patients was 7.92% (standard deviation = 1.79%). Overall, 35% of the patients had glycemic level under control.  Table 1 shows that sociodemographic characteristics in patients who had HbA1c under control versus those not under control were similar, with only incomes differing significantly between the two groups.  Table 2 presents the descriptive statistics of the key measurements in the pathway analysis followed.

### 3.2. Correlations between Social Support, Adherence, Self-Efficacy, and Hb1A1c

Table 3 presents the pairwise Pearson's correlations between the four study variables, where HbA1c was treated as continuous variables. Total adherence and diet adherence were correlated significantly with objective support; diet adherence and lifestyle adherence were correlated significantly with self-efficacy; and self-efficacy were correlated significantly with subjective social support and support utilization. As expected, social support, adherence, and self-efficacy were negatively associated with HbA1c levels.

### 3.3. The Mediating Effects of Self-Efficacy and Adherence on the Association between Social Support and Glycemic Control

**Figure 1:**
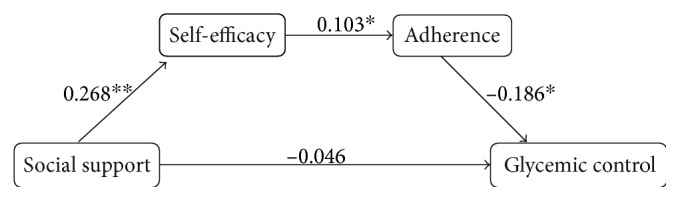
Pathway between social support and glycemic control, mediated by self-efficacy and adherence. Overall model goodness-of-fit statistics: *χ*^2^ = 2.47, *P* = 0.12; GFI = 0.99; AGFi = 0.98; CFI = 0.98; RMSEA = 0.05. ^∗∗^*P* < 0.01; ^∗^*P* < 0.05.

Table 4 summarizes the regression results exploring the pathway from social support to glycemic control through self-efficacy and adherence, as illustrated in Figure 1. The first set of three regressions (i.e., the first pathway) shows that, controlling for confounding variables (i.e., variables in Table 1), social support had significant effects on self-efficacy and glycemic control when analyzed separately, but social support's effect on glycemic control became insignificant when self-efficacy was controlled, indicating that social support's effect on glycemic control was completely mediated by social support's effect on self-efficacy. Similarly, the second set of regressions indicates that social support's effect on glycemic control was completely mediated through its effect on adherence, when self-efficacy was not taken into consideration. The third set of regressions shows that, along the path, self-efficacy also had significant effect on glycemic control, and that effect was partially mediated by adherence since both adherence and glycemic control were significant when both entered into the regression. Using the formula by MacKinnon et al. [26], the size of the mediating effect of adherence on the relationship between self-efficacy was 16.63% (i.e., 0.106 × −0.171/−0.109 = 16.63%).

Figure 1 depicts the structural equation model estimated by SPSS AMOS that directly examined our hypothesized pathway from social support to glycemic control. The path coefficients for the three paths are statistically significant, while the direct path from social support to glycemic control is not significant. The goodness-of-fit indices (*χ*^2^ = 2.47, *P* = 0.12; GFI = 0.99; AGFi = 0.98; CFI = 0.98; and RMSEA = 0.05) indicate a strong fit between our data and the hypothesized pathway. Overall, the results showed that social support had a direct effect on self-efficacy, which had a direct effect on adherence, which, in turn, had a direct effect on glycemic control. When the effects of self-efficacy and adherence were counted for, social support had no direct effect on glycemic control. In other words, social support's effect on glycemic control was completely mediated by self-efficacy and adherence.

## 4. Discussion

Our data suggests that only a small percentage (35%) of T2DM patients achieved adequate glycemic control, consistent with the findings by other researchers from China and around the world [24, 28]. Our findings also add to the previous evidence that self-efficacy, adherence, and social support, individually and together, have significant effects on glycemic control [29–31].

The primary findings of this study confirm our hypothesis that social support significantly affects glycemic control and the effect is mediated by self-efficacy and adherence. Both hierarchical regressions and structural equation modeling analyses suggest that greater social support may strengthen a patient's self-efficacy, which, in turns, translates into better adherence, which, in turn, results in improved glycemic control in T2DM patients. Our results also showed that social support's effect on glycemic control is completely explained by this pathway: when the mediating effects of self-efficacy and adherence are accounted for, social support has no direct effect on glycemic control.

Many researchers have examined the interactions between social support, self-efficacy, and adherence in patients with T2DM and other chronic diseases and analyzed their independent and correlated effects on patient outcomes. For example, Schiøtz et al. showed that frequent contact with family and friends was associated with more positive scores for activation (i.e., self-efficacy) and health-promoting self-management behavior, such as frequent exercising and frequent foot examinations in T2DM patients, and that a poor functional social network, measured as perceived lack of help in the event of severe illness, was associated with low patient activation [32]. Kanbara et al. found that augmentation of emotional support to patients significantly increased the active coping behavior [33]. Many other researchers reported similar results, showing the positive associations between social support, self-efficacy, and positive patient behavior (e.g., adherence) based on the data from various settings and patient populations [34–37]. But some researchers reported insignificant, inconsistent results, even results opposite to expectations [11, 12, 17, 38–40]. Taken together, these previous studies have increased our understanding of the importance of these factors in improving the patient outcomes of T2DM patients and other patients, but the relationship remains rather tangled and unclear.

Our study was built upon our understanding that social support, being an environment factor external to a patient, could become internalized to induce changes in the patient's attitude toward his or her medical conditions, which, then, could lead to behavior changes in the forms of adherence and other coping behaviors. Stronger social support from family, friends, and communities could cultivate positive mental and emotional changes within a patient; strengthens his resolve, belief, and confidence in managing his or her conditions; and improving his or her quality of life. Such internal change, that is, improved self-efficacy, is necessary for a patient to maintain sustained, positive behavior changes, to adhere to treatment regiment, among other things. Overall, our data suggest that social support has a direct effect in a patient's self-motivation and confidence in managing his or her diabetes, and this improved self-efficacy could translate into improved medical adherence, which results in improved glycemic control. It is also likely that social support directly affects glycemic control, for example, a husband reminding his wife to take medication or a wife keeping a close watch of her husband's diet, but our data shows that such effects may be quite small or insignificant when social support's strong impact on self-efficacy is accounted for.

This study has some limitations. First, the generalizability of our findings is limited since our sample was small and all participants came from only two hospitals. In other words, our participants are not representative of the broader population of T2DM patients in China. Second, we could not make causality inferences between these variables of interest because we only had cross-sectional data. Third, our findings might suffer from self-report bias, which is common to studies based on data collected from self-completed questionnaires. Fourth, there was also likely bias due to the fact that patients who refused to participate were automatically excluded from the study; because we were not able to collect data from them, such bias could not be assessed. However, since our purpose was to investigate the cross-sectional associations between four variables, and we used both classical statistical methods and more advanced pathway analyses to examine the mediating effects along the pathway, these limitations should not be serious enough to threaten the hypothesis testing results of our study.

In conclusion, our study confirmed the role of social support in T2DM management and clarified the mediating roles of self-efficacy and adherence along the pathway from social support to glycemic control. Our findings indicate that social support must be recognized as a key element in any intervention aimed at improving glycemic control in T2DM patients.

## Figures and Tables

**Table 1:** Patient characteristics by level of glycemic control.

Variables	Glycemic control = yes	Glycemic control = no	*P*
	*N* = 186	*N* = 346	
Male (*n*, %)	78, 41.9%	146, 42.2%	0.900
Age (mean ± SD)	63.27 ± 10.95	63.51 ± 11.19	0.742
Inpatient (*n*, %)	41, 22.0%	93, 26.9%	0.250
Educational level (*n*, %)			0.060
Primary school or lower	47, 25.3%	97, 28.0%	
Middle school	31, 16.7%	79, 22.8%	
High school	56, 30.1%	99, 28.6%	
University/college or higher	52, 28.0%	71, 20.5%	
Individual income (monthly, ¥, *n*, %)			0.041^∗^
<1000	57, 30.6%	80, 23.1%	
1000–1999	70, 37.6%	129, 37.3%	
2000–2999	26, 14.0%	60, 17.3%	
3000–3999	18, 9.7%	37, 10.7%	
≥4000	15, 8.1%	40, 11.6%	
Family size (*n*, %)			0.815
1 persons	8, 4.3%	20, 5.8%	
2-3 persons	102, 54.8%	184, 53.2%	
4-5 persons	60, 32.3%	107, 30.9%	
≥6 persons	16, 8.6%	35, 10.1%	
Smoking (*n*, %)			0.622
Never	164, 88.2%	299, 86.4%	
Occasionally	5, 2.7%	12, 3.5%	
Regular	17, 9.1%	35, 10.1%	
Drinking (*n*, %)			0.597
Never	161, 86.6%	300, 86.7%	
Occasionally	17, 9.1%	38, 11.0%	
Regular	8, 4.3%	8, 2.3%	
Exercising (*n*, %)			0.359
Never	27, 14.5%	58, 16.8%	
Occasionally	44, 23.7%	88, 25.4%	
Regular	115, 61.8%	200, 57.8%	

Monthly individual income, in Chinese Yuan; ^∗^*P* < 0.05.

**Table 2:** Descriptive statistics of social support, self-efficacy, adherence, and HbA1c.

Variables	$\overset{¯}{x}\pm s$
Social support	37.00 ± 7.73
Objective support	8.62 ± 3.27
Subjective support	21.65 ± 4.96
Supportive utilization	6.74 ± 2.42
Total adherence	33.63 ± 3.28
Medication adherence	10.84 ± 1.78
Diet adherence	9.45 ± 2.27
Lifestyle adherence	10.74 ± 1.43
Self-efficacy	38.60 ± 11.15
HbA1c (%)	7.92 ± 1.79

**Table 3:** Pairwise correlations between social support, adherence, self-efficacy, and HbA1c level.

	1	1a	1b	1c	2	2a	2b	2c	3	4
(1) Social support										
(1a) Objective support	0.652^∗∗^									
(1b) Subjective support	0.871^∗∗^	0.351^∗∗^								
(1c) Supportive utilization	0.484^∗∗^	0.049	0.254^∗∗^							
(2) Total adherence	0.067	0.102^∗^	0.014	0.077						
(2a) Medication	−0.030	−0.003	−0.022	0.018	0.762^∗∗^					
(2b) Diet	0.156^∗∗^	0.139^∗∗^	0.138^∗∗^	0.023	0.284^∗∗^	0.309^∗∗^				
(2c) Lifestyle	0.081	0.010	0.075	0.127^∗∗^	0.182^∗∗^	0.151^∗∗^	0.156^∗∗^			
(3) Self-efficacy	0.277^∗∗^	0.064	0.305^∗∗^	0.169^∗∗^	0.078	0.081	0.145^∗∗^	0.273^∗∗^		
(4) HbA1c	−0.092^∗^	−0.037	−0.095^∗^	−0.035	−0.150^∗∗^	−0.127^∗∗^	−0.143^∗∗^	−0.057	−0.146^∗∗^	

^∗∗^ *P* < 0.01; ^∗^*P* < 0.05.

**Table 4:** Standardized coefficients indicating the mediating effects of self-efficacy and adherence on the relationship between social support and glycemic control.

Pathway	Step (a)	Step (b)	Step (c)
SS→SE→GC	GC = −0.088^∗^SS	SE = 0.265^∗∗^SS	GC = −0.063SS − 0.092^∗^SE
SS→Ad→GC	GC = −0.088^∗^SS	Ad = 0.101^∗^SS	GC = −0.070SS − 0.173^∗∗^Ad
SE→Ad→GC	GC = −0.109^∗^SE	Ad = 0.106^∗^SE	GC = −0.091^∗^SE − 0.171^∗∗^Ad

SS: social support; SE: self-efficacy; Ad: adherence; GC: glycemic control; ^∗∗^*P* < 0.01; ^∗^*P* < 0.05.
